# Gut microbiota of adults with asthma is broadly similar to non-asthmatics in a large population with varied ethnic origins

**DOI:** 10.1080/19490976.2021.1995279

**Published:** 2021-11-08

**Authors:** Robert F. J. Kullberg, Bastiaan W. Haak, Mahmoud I. Abdel-Aziz, Mark Davids, Floor Hugenholtz, Max Nieuwdorp, Henrike Galenkamp, Maria Prins, Anke H. Maitland-van der Zee, W. Joost Wiersinga

**Affiliations:** a Center for Experimental and Molecular Medicine (CEMM), Amsterdam University Medical Centers - Location Amc, University of Amsterdam, Amsterdam, The Netherlands; b Microbiota Center Amsterdam, Amsterdam University Medical Centers - Location Amc, University of Amsterdam, Amsterdam, The Netherlands; c Department of Respiratory Medicine, Amsterdam University Medical Centers - Location Amc, University of Amsterdam, Amsterdam, The Netherlands; d Amsterdam Diabetes Center, Department of Internal Medicine, Amsterdam University Medical Centers, Academic Medical Center, Vu University Medical Center, Amsterdam, The Netherlands; e Wallenberg Laboratory, Sahlgrenska Academy at University of Gothenburg, Gothenburg, Sweden; f Department of Vascular Medicine, Amsterdam University Medical Centers - Location Amc, University of Amsterdam, Amsterdam, The Netherlands; g Department of Public Health, Academic Medical Center, University of Amsterdam, Amsterdam, The Netherlands; h Department of Infectious Diseases, Public Health Service of Amsterdam, Amsterdam, The Netherlands; i Department of Internal Medicine, Division of Infectious Diseases, Amsterdam University Medical Centers - Location Amc, University of Amsterdam, Amsterdam, The Netherlands

**Keywords:** Asthma, microbiome, bacterial community composition, gastrointestinal tract, adults, ethnicity, HELIUS study

## Abstract

Bacterial gut communities might predispose children to develop asthma. Yet, little is known about the role of these micro-organisms in adult asthmatics. We aimed to profile the relationship between fecal microbiota and asthma in a large-scale, ethnically diverse, observational cohort of adults. Fecal microbiota composition of 1632 adults (172 asthmatics and 1460 non-asthmatics) was analyzed using 16S ribosomal RNA gene sequencing. Using extremely randomized trees machine learning models, we assessed the discriminatory ability of gut bacterial features to identify asthmatics from non-asthmatics. Asthma contributed 0.019% to interindividual dissimilarities in intestinal microbiota composition, which was not significant (*P* = .97). Asthmatics could not be distinguished from non-asthmatics based on individual microbiota composition by an extremely randomized trees classifier model (area under the receiver operating characteristic curve = 0.54). In conclusion, there were no prominent differences in fecal microbiota composition in adult asthmatics when compared to non-asthmatics in an urban, large-sized and ethnically diverse cohort.

## Introduction

Asthma is one of the most common chronic respiratory diseases, as over 300 million people have asthma-related symptoms.^1^ Emerging evidence indicates that intestinal microbiota are involved in development of asthma in children.^1^ For example, infants with an immature microbial composition have an increased risk of developing asthma at 5 years of age and disrupted neonatal gut microbiota might promote CD4^+^ T-cell dysfunction thereby increasing susceptibility to childhood allergic asthma.^2,3^ Although these findings provide clues that gut microbiota determine the risk of asthma development in children, little is known about the role of these micro-organisms in adult asthmatics. Gut bacteria might reconstitute in later life – having already produced life-long effects on the immune system during a ‘window of opportunity’ in early life – and may no longer be involved in adults. Results of the few studies addressing this role are conflicting, describing associations between adult asthmatics and specific gut bacteria that are not confirmed by others.^4–9^ These differences might be due to low sample size (the largest study included 158 cases and several studies had less than 40 asthmatics), incomplete confounder analysis (e.g. ethnicity) or difficulties in analyzing microbiota data.^10^

In this study, we use data from the large and ethnically diverse Healthy Life in an Urban Setting (HELIUS) study,^11,12^ classical microbial ecology and extremely randomized trees-based machine learning, and show that there is no evidence of an important relationship between individual differences in 16S rRNA gene sequencing-based microbiota composition and asthma in adults.

## Results

The HELIUS study is a large multi-ethnic cohort study of Amsterdam (The Netherlands) residents who were randomly sampled, stratified by ethnic origin. By using a large, urban-based and ethnically diverse cohort, we were able to control for several important confounders including, among others, ethnicity and the recent use of antibiotics (see Patients and methods). After exclusion of participants with a smoking history of more than 10 pack years to avoid potential confusion with chronic obstructive pulmonary disease or other smoking related complaints (n = 460), 1632 participants of the HELIUS study who donated stool samples were included in this study.^11,12^ Of 1632 participants, 172 (10.5%) were considered asthmatics based on doctors’ diagnosis as self-reported. Participants without self-reported doctors’ diagnosis of asthma were considered non-asthmatics (n = 1460). There were no statistically significant differences between asthmatics and non-asthmatics in their age, smoking history, recent antibiotic or probiotic use or season of sample collection (Table 1).

**Table 1.** Baseline characteristics of included HELIUS participants

	Asthma	Non-asthmatics	
	(n = 172)	(n = 1460)	*P* value
**Sex, no (%)**			<0.001
Men	56 (33)	630 (43)	
Women	116 (67)	830 (57)	
**Age (yrs), mean (SD)**	49.7 (11.4)	50.1 (11.1)	0.490
**Ethnicity^a^, no (%)**			<0.001
Dutch	26 (15)	262 (18)	
Surinamese	70 (41)	603 (41)	
Turkish	23 (13)	111 (8)	
Moroccan	33 (19)	187 (13)	
Ghanaian	20 (12)	293 (20)	
Other	0 (0)	4 (0)	
**BMI, mean (SD)**	28.7 (5.8)	27.4 (4.8)	0.001
**Smoking status, no (%)**			0.213
Yes	16 (9)	133 (9)	
Never	125 (73)	1078 (74)	
Stopped	31 (18)	249 (17)	
**Uses alcohol^b^, no (%)**	76 (44)	723 (50)	0.340
**Diet^c^**			
Total fatty acids (grams per day), mean (SD)	71.8 (40.2)	79.0 (41.3)	0.016
Saturated fatty acids (grams per day), mean (SD)	25.4 (17.4)	26.6 (15.8)	0.086
Fibers (grams per day), mean (SD)	23.6 (10.1)	24.6 (11.2)	0.125
**Recent antibiotics^d^, no (%)**	18 (11)	129 (9)	0.450
**Current use of probiotics, no (%)**	7 (4)	78 (5)	0.227
**Current use of corticosteroids, no (%)**	20 (12)	66 (5)	<0.001
**Season of sample collection**			0.323
Spring	31 (18)	290 (20)	
Summer	55 (32)	464 (32)	
Autumn	52 (30)	360 (25)	
Winter	34 (20)	346 (24)	

a. Based on country of birth: participants were considered of non-Dutch origin if they were born outside the Netherlands and had at least one parent who was born outside the Netherlands; or if they were born in the Netherlands but both parents were born outside the Netherlands.

b. Use of alcoholic beverages in the past 12 months.

c. Detailed information on diet was obtained from 47% of the participants.

d. Use of antibiotics in the 3 months prior to fecal sample collection.

To assess the impact of the used definition of asthma, we repeated all analyses using two different asthma definitions. First, a strict definition was used where participants were considered asthmatics solely based on medication use. Second, a broader definition was used and asthmatics were identified based on self-reported symptoms, medication and/or doctors’ diagnosis. 94 participants (5.8%) had asthma based on their medication use. 358 (21.9%) were considered asthmatics based on the broader definition.

**Figure 1.**
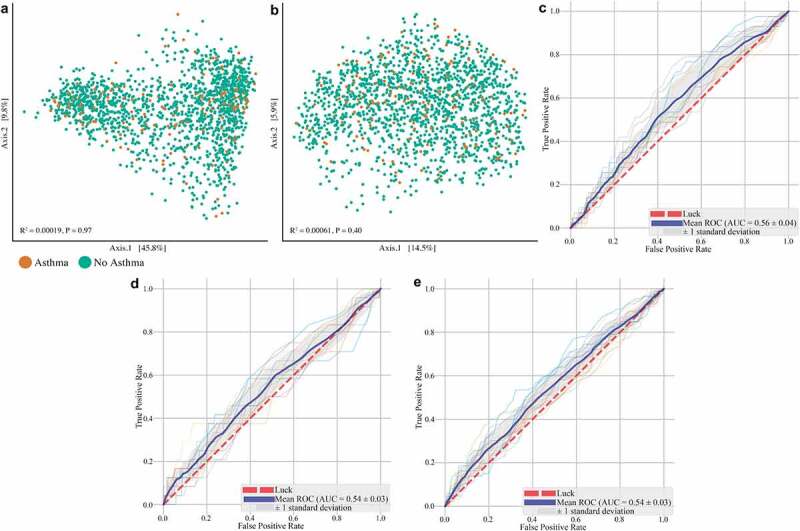
**Marginal contribution of asthma to interindividual dissimilarities in microbiota composition**. Differences in intestinal microbiota β-diversity with weighted (a) and unweighted Unifrac distance (b) of adult asthmatics (n = 172) compared to non-asthmatics (n = 1460). Receiver operating characteristic curve of the extremely randomized trees classifier showing asthmatics could not be distinguished from non-asthmatics based on individual microbiota composition (c). This was not changed with two alternative definitions for asthma: participants were considered asthmatics based on use of asthma medication (d) or based on self-reported symptoms, medication use and/or doctors’ diagnosis (e). ROC = receiver operating characteristic; AUC = area under the curve

Fecal microbiota composition was profiled by sequencing the 16S rRNA gene, V4 region (Patients and methods). As earlier described,^12,13^ Firmicutes and Bacteroidetes were the dominant phyla, and the most abundant families belonged to these phyla (e.g. Lachnospiraceae, Ruminococcaceae, Bacteroidaceae) (Supplementary Figure 1). Asthma contributed 0.019% to interindividual dissimilarities in intestinal microbiota composition, which was not significant (*P* = .97; Table 2). Consequently, no distinct clusters were observed when visualizing ß-diversity by principal coordinate analysis (Figure 1a,b). When we controlled for potential confounders (participant characteristics, recent antibiotic use, sample collection seasonality, dietary fiber/fat, usage of probiotics and corticosteroids) in a multivariable analysis, the variance explained by asthma was not meaningfully changed (R^2^ = 0.00016; Table 2). The contribution of asthma to interindividual dissimilarities was small compared to other determinants, as all tested variables together explained 7.2% of the interindividual dissimilarities in microbiota composition. Asthma contributed only 0.22% to this total of 7.2% in interindividual dissimilarities (0.00016 from a total R^2^ of 0.072; Table 2) while ethnicity contributed 42.6% and sex 31.6%, indicating that the gut microbiota of asthmatics is broadly similar to those of non-asthmatics. The use of stricter and broader definitions for asthma did not meaningfully impact these results (Supplementary Table 1). When we excluded participants with comorbidities (diabetes mellitus, hypertension, cardiovascular disease, chronic gastrointestinal disease, malignancy, stroke and rheumatic diseases), we found a comparable contribution of asthma to interindividual differences in microbiota composition (0.058%, *P* = .71; Supplementary Table 2).

**Table 2.** Univariable and multivariable analysis of associations between individual characteristics and gut microbiota β-diversity of asthmatics vs. non-asthmatics

	**Weighted Unifrac**				**Weighted Unifrac**				
	Univariable – adonis				Multivariable – PERMANOVA				
	Df	R^2^	F	*P* value	Df	R^2^	F	*P* value	Contribution to total inter-individual dissimilarities
**Asthma**	1	0.00019	0.312	0.965	1	0.00016	0.287	0.970	0.22%
**Sex**	1	0.02392	39.89	0.001	1	0.02289	40.54	0.001	31.60%
**Age**	1	0.00303	4.943	0.001	1	0.00179	3.162	0.016	2.47%
**Ethnicity**	5	0.05363	18.41	0.001	5	0.03089	10.94	0.001	42.64%
**BMI**	1	0.00872	14.33	0.001	1	0.00587	10.39	0.001	8.10%
**Smoking status**	2	0.00398	3.254	0.005	2	0.00191	1.695	0.082	2.64%
**Alcohol use**	1	0.00831	6.814	0.001	1	0.00115	1.017	0.388	1.59%
**Total fatty acids**	1	0.00454	7.430	0.001	1	0.00046	0.813	0.486	0.64%
**Fibers**	1	0.00416	6.797	0.002	1	0.00108	1.914	0.084	1.49%
**Saturated fatty acids**	1	0.00411	6.719	0.001	1	0.00036	0.629	0.700	0.50%
**Recent antibiotics**	1	0.00226	1.845	0.058	1	0.00193	1.712	0.058	2.66%
**Use of probiotics**	1	0.00150	1.222	0.239	1	0.00062	0.548	0.893	0.86%
**Use of corticosteroids**	1	0.00064	1.041	0.325	1	0.00055	0.982	0.362	0.76%
**Season of sample collection**	3	0.00366	1.991	0.019	3	0.00278	1.644	0.055	3.84%
**Total**						0.07244			100%

Analyses were performed by permutational multivariate analysis of variance (PERMANOVA) with the Weighted UniFrac distance. The multivariable model includes sex, age, ethnicity, BMI, smoking status, alcohol consumption, dietary variables (total fatty acids, saturated fatty acids and fibers), recent use of antibiotics (3 months prior to fecal sample collection), usage of probiotics and corticosteroids, and the season of sample collection. *Df*: degrees of freedom.

Next, to investigate if differences in intestinal microbiota composition existed on an individual bacterial taxon level, we used extremely randomized trees-based machine learning models (Patients and methods). Asthmatic participants could not be distinguished from non-asthmatic participants based on their individual microbiota composition by this model (area under the receiver operating characteristic curve (AUC-ROC) = 0.56 ± 0.04; Figure 1c). When we used other definitions for asthma, the AUC-ROC was not meaningfully changed (Figure 1d,e), nor did the use of two different machine learning approaches (random forest and support vector machines) alter our findings (Supplementary Figure 3).

**Figure 2.**
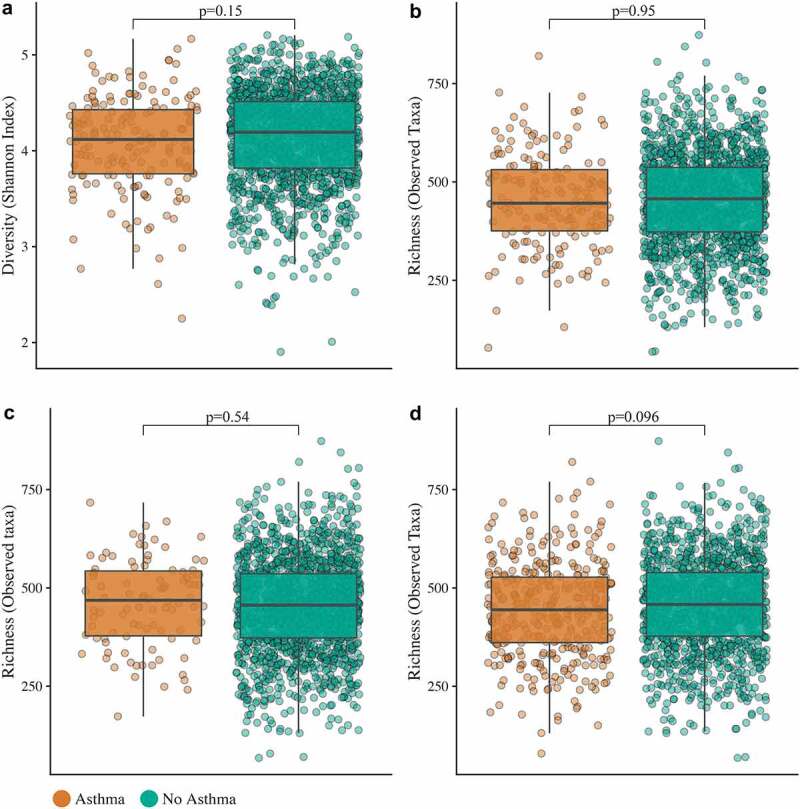
**Insignificant differences in microbiota richness and Shannon α-diversity between asthmatics and controls**. No significant differences in Shannon diversity (a) and richness (b) between adult asthmatics (n = 172) and non-asthmatics (n = 1460)

In addition, no differential abundant taxa between asthmatics and non-asthmatic participants were identified (Holm corrected *p*-values ≥ 0.05) using a DESeq2 model both with and without covariates (participant characteristics, recent antibiotic use, sample collection seasonality, usage of probiotics and corticosteroids). Furthermore, there were no differences in microbiota richness or Shannon alpha diversity between asthmatic and non-asthmatic participants (Figure 2).

No differences in richness when participants were considered asthmatics based on use of asthma medication (stricter definition) (C) or based on self-reported symptoms, medication use and/or doctors’ diagnosis (broader definition) (D). In the box plots the central line shows the median, the box limits are the first and third quartile, and whiskers above and below the box.

Following exclusion of participants with comorbidities (diabetes mellitus, hypertension, cardiovascular disease, chronic gastrointestinal disease, malignancy, stroke and rheumatic diseases), our extremely randomized trees model had no capacity to discriminate asthmatics from non-asthmatics (AUC-ROC = 0.56 ± 0.04; Supplementary Figure 2), no differential abundant taxa between asthmatics and non-asthmatic participants were identified (Holm corrected *p*-values ≥ 0.05) and we found no differences in microbiota richness or Shannon alpha diversity between asthmatic and non-asthmatic participants (Supplementary Figure 2).

Finally, we asked if the composition of the intestinal microbiota is associated with symptom control in asthma. We compared asthmatics with self-reported asthma complaints (attack of shortness of breath with wheezing) or use of asthma medication in the past year (n = 101), to asthmatics without complaints or medication usage in the past year (n = 71). We observed no differences in microbiota richness (*P* = .99), α-diversity (*P* = .6) or β-diversity (*P* = .906) between these groups (Supplementary Figure 4). Thus, these results suggest that there is no relationship between the composition of the intestinal microbiota, based on 16S rRNA gene sequencing, and the reported control of asthma symptoms in adults.

## Discussion

Despite multiple studies showing the involvement of intestinal microbiota in asthma development in children,^1–3^ little is known about the relationship between intestinal microbiota and asthma in adults. Here, we show that there are no prominent differences in 16S rRNA gene sequencing-based fecal microbiota composition in adult asthmatics when compared to non-asthmatics in an urban, large-sized and ethnically diverse cohort.

Asthma contributed only 0.019% to interindividual dissimilarities in gut microbiota composition which is marginal – especially when compared with other determinants such as sex (3.03%) and ethnicity (5.36%). Our extremely randomized trees had no capacity to discriminate asthmatics from non-asthmatics based on intestinal microbiota composition, which is in line with an earlier described random forest classifier model that had little value in predicting asthma (AUC-ROC = 0.53),^6^ and further supports a negligible contribution of asthma to interindividual dissimilarities in 16S rRNA gene sequencing based microbiota composition.

Earlier studies provided conflicting results on differential abundant taxa in adult asthmatics. Hevia and colleagues^5^ reported a higher abundance of, among others, *Faecalibacterium* species in asthmatics, while Wang et al^4^ reported lower abundances of these species. Gut bacteria might reconstitute in later life -having already produced life-long effects on the immune system during a ‘window of opportunity’ in early life- and may no longer be involved in adults when the microbiota and immune system have matured.^14,15^ In addition, fecal microbiota might not be the only culprit in asthma, as T-cells – which are related to asthma development^16^ – are trained by microbiota in the small intestine.^17^ Thus, further studies will have to show if small intestine microbiota are associated with asthma at different points in life.

To the best of our knowledge, this is the largest cohort study of its kind in the field which enabled us to control for several important confounders. However, our approach has limitations. First, asthmatics were not identified by demonstration of reversible airway obstruction using spirometry or airway hyperresponsiveness,^18^ which may have led to incorrect classification. However, additional analyses with broader and more strict criteria for asthma did not alter our findings. Second, asthma is considered an umbrella term for different phenotypes that arise through different pathophysiologic pathways.^1^ We cannot fully exclude a role of fecal bacteria in a certain phenotype. Finally, the taxonomic resolution at species level of 16S rRNA gene sequencing is limited. Increasing the resolution using metagenomic sequencing might reveal differences in diversity and provide greater insight into functional capacities of the microbiome.

In conclusion, while emerging evidence indicates that shifts in intestinal microbiota composition influence the risk of developing asthma in children, we found no prominent differences in fecal microbiota composition in adult asthmatics when compared to non-asthmatics in an urban, large-sized and ethnically diverse cohort. Future investigations should focus on the role of fecal bacteria in specific asthma phenotypes or on other potential mechanisms, such as an age-dependent effect of gut microbiota.

## Patients and methods

### Participants

Participants were recruited as part of the HELIUS study, a large multi-ethnic cohort study of Amsterdam residents (aged 18–70). Details of recruitment, dietary information, fecal sampling and microbiota sequencing have been previously published.^11,12,19^ In brief, the HELIUS study is a multi-ethnic cohort study conducted in Amsterdam, The Netherlands. Between 2011 and 2015, subjects (aged 18–70) were randomly, stratified by ethnicity, selected from the municipal registry of Amsterdam. Information on sociodemographic characteristics, lifestyle (including smoking, alcohol use, diet) and history of diseases was gathered through a questionnaire (including questions on asthma complaints), a physical examination (including, among other things, height and weight) and by bringing all currently used medications to the research site.

Of 22165 participants who completed the questionnaire and who took part in the physical examination, 5927 provided stool samples. This study includes data obtained on the first 2170 feces samples that were collected and processed.^12^ The sample size was chosen based on a previously published analysis of gut microbiome variation and on earlier work by our study consortium where we also used data from the HELIUS study and observed numerous differences of small magnitude in microbiota composition that were significant.^12,20^

For the purposes of this study, participants with a smoking history of more than 10 pack years were excluded to avoid potential confusion with chronic obstructive pulmonary disease or smoking related complaints. Asthmatics were identified based on a self-reported doctors’ diagnosis of asthma (*“Has a doctor ever diagnosed you with asthma?’*). The reference group consisted of participants with a negative answer to this question.

To examine the influence of our definition of asthma, we repeated all analyses using two different definitions to identify asthmatics aimed at capturing a more strict and a broader group of asthmatics. First, participants were considered asthmatics if they used asthma (or COPD) medication (ATC-code R03) and the reference group consisted of those participants who did not use such medication. Second, participants were considered asthmatics based on (1) a doctors’ diagnosis of asthma and/or (2) use of asthma (or COPD) medication (ATC-code R03) and/or (3) self-reported asthma symptoms (“*Have you ever had an attack of shortness of breath with wheezing (noisy breathing)?*”). The reference group consisted of participants with a negative answer given to all of the three above mentioned questions.

Participants with missing information on doctors’ diagnosis of asthma, use of asthma medication or self-reported asthma symptoms were excluded (n = 16). The final study population consisted of 1632 participants.

As previously described,^12,21,22^ detailed dietary information was collected on a subsample of HELIUS participants through ethnic-specific semiquantitative food frequency questionnaires with a reference period of four weeks. Detailed dietary information was available for 47% of the participants included in the analyses of the current study (n = 767).

Written informed consent was obtained from all participants. Ethical approval for the HELIUS study was received from the Medical Ethics Committee of the Academic Medical Center (protocol number: 10/100; amendment 10/100#10.17.1729; NL32251.018.10) and all research was conducted in accordance with the declaration of Helsinki.

### Microbiota sequencing and analysis

Stool sample collection and microbiota sequencing was performed as described in detail elsewhere.^12,23^ Briefly, participants were asked to bring a ‘fresh’ stool sample to the research location within 6 hours after collection. If not possible, they were instructed to keep the stool sample in their freezer overnight and bring it in frozen to the research location the next morning. At the research location, samples were temporarily stored at −20°C until daily transportation and storage at −80°C. No information was recorded on whether samples arrived either frozen or fresh at the research location.

Stool samples were shipped to the Wallenberg Laboratory (Sahlgrenska Academy at University of Gothenburg, Sweden) to determine the fecal microbiota. DNA was extracted using a repeated bead beating method. To profile the composition of microbiota, the V4 region of the 16S rRNA gene was sequenced (Illumina MiSeq). Negative controls were included for each sample and gel electrophoresis was used to confirm the absence of detectable PCR products in these negative controls. Positive controls were not included in these runs but the protocol used to analyze the samples was optimized using mock samples. Raw sequencing reads were processed with USEARCH (v11.0.667).^24^ Paired-end reads were merged, allowing one expected error in the merged contig and a maximum of 30 differences in the overlapping region. We performed expected error-based read quality filtering as described by Edgar et al.^25^ The remaining contigs were dereplicated and subsequently denoised to infer Amplicon Sequence Variants (ASVs) using the UNOISE3 algorithm.^25^ A count table was produced by mapping all merged reads against the resulting ASVs. ASVs not matching expected amplicon length (shorter than 250 bp or longer than 260 bp) were removed. The DADA2 package (function ‘assignTaxonomy) and SILVA reference database (version 132) were used to assign taxonomy.^26,27^ ASVs sequences were aligned using MAFFT with auto settings.^28^ A phylogenetic tree was constructed from the resulting multiple sequence alignment using a generalized time-reversible model with the ‘double-precision’ build of FastTree (version 2.1.11).^29^ The ASV table was rarefied to 14894 counts per sample and integrated with the taxonomy and tree using the phyloseq package.^30^

### Statistical analysis

All analyses, except the extremely randomized trees classifier, were performed using R (version 3.6.0). Two-tailed level of significance between groups was set at *P* < .05. β-diversity was assessed using the weighted and unweighted Unifrac distance using principal coordinates analysis with the phyloseq package.^30^ PERMANOVA models (vegan package,^31^ functions Adonis and Adonis2, 999 permutations) were used to assess the contribution of asthma and the following potential confounders to interindividual dissimilarities in intestinal microbiota composition (β-diversity using the weighted UniFrac distance which takes the bacterial phylogeny into account): sex, age, ethnicity, BMI, smoking status, alcohol consumption, dietary variables (total fatty acids, saturated fatty acids and fibers), recent use of antibiotics (3 months prior to fecal sample collection), usage of (over-the-counter) probiotics and corticosteroids (ATC codes D07 plus specific codes), and the season of sample collection. These models decompose the dissimilarity matrix into ‘variance’ explained by each variable. The obtained R^2^ gives the proportion of variability explained by a certain variable, as percentage of the entire dissimilarity.

Extremely randomized trees classifier analysis was used to assess the value of the intestinal microbiota to distinguish asthmatic participants from non-asthmatics, using the sklearn package and python version 3.8.1.^32^ We used count data of ASVs that were present at greater than 10% of the sample population or a minimum abundance of 500 counts, which resulted in 1381 ASVs as input to the extremely randomized trees classifier analysis. We performed 100-iterations of 5-fold cross validation on 75% of the dataset, with subsequent testing on the remaining 25% of the samples, and subsequently assessed the performance of the microbiota by calculating the mean area under the receiver operating characteristic curve of all 20 shuffles.

In addition, we used a random forest ensemble learning approach (randomForest package^33^) and Support Vector Machines as implemented in the caret^34^ and kernel^35^ packages with a polynomial kernel. For model creation, we used count data of ASVs that were present at present at greater than 10% of the sample population. For the random forest models, we performed 20-fold random splitting of the dataset into a training (75%) and validation (25%) set and trained the models using the training data with 501 trees and the proximity parameter set to TRUE. Default settings were used for all other parameters. For the Support Vector Machines, we performed 5-fold splitting into a training (75%) and validation (25%) set, and trained the models with 5-fold cross validation. Hyperparameter tuning was automatically executed by caret via grid search (C: 0.01, 0.05, 0.1, 0.25, 0.5, 0.75, 1.0, 1.25, 1.5, 1.75, 2, 5; Scale: 0.01, 0.03, 0.06, 0.09; Degree: 0, 1, 2, 3, 4, 5). Subsequently, both the random forest models and Support Vector Machines models were tested by calculating the area under the receiver operating characteristic curve (AUC-ROC) using the pROC package on the remaining 25% of the samples.

Alpha diversity was assessed by calculating Observed Taxa Richness index and Shannon Diversity Index with the phyloseq package.^30^ The ‘DESeq’ function in DESeq2 was used to test for differentially abundant taxa between asthmatics and non-asthmatics, with Holm correction for multiple comparisons.^36^ We restricted our DESeq analysis to ASVs that were present at greater than 10% of the sample population.

## Supplementary Material

Supplemental Material

## Data Availability

The 16S rRNA gene sequences have been deposited in the European Genome-phenome Archive (accession number EGAD00001004106). The HELIUS data are owned by the AMC in Amsterdam, The Netherlands. Any researcher can request the data by submitting a proposal to the HELIUS Executive Board as outlined at http://www.heliusstudy.nl/en/researchers/collaboration and data will be provided upon reasonable request and in line with current GDPR legislation.
