# Supplementary material for: Gut microbiota of adults with asthma is broadly similar to non-asthmatics in a large population with varied ethnic origins
Source: Gut Microbes. 2021 Nov 8;13(1):1995279. doi: 10.1080/19490976.2021.1995279 (PMC8583066; doi:10.1080/19490976.2021.1995279)
Supplement: Supplemental Material [file KGMI_A_1995279_SM2235.docx]

**Supplementary Material**

**Gut microbiota of adults with asthma is broadly similar to non-asthmatics in a large population with varied ethnic origins**

Robert F.J. Kullberg, MD, Bastiaan W. Haak, MD PhD, Mahmoud I. Abdel-Aziz, MSc, Mark Davids, MSc, Floor Hugenholtz, PhD, Max Nieuwdorp, MD PhD, Henrike Galenkamp, PhD, Maria Prins, PhD, Anke H. Maitland-van der Zee, PhD, W. Joost Wiersinga, MD PhD

**Supplementary Table 1. Alternative asthma definitions for univariable and multivariable analysis of associations between individual characteristics and β-diversity of asthmatics vs. controls**

|  | **(1) Medication based asthma - Weighted Unifrac** | | | | | | | | **(2) Asthma based on self-reported symptoms, medication and/or doctors’ diagnosis - Weighted Unifrac** | | | | | | | |
| --- | --- | --- | --- | --- | --- | --- | --- | --- | --- | --- | --- | --- | --- | --- | --- | --- |
|  | Univariable - adonis | | | | Multivariable - PERMANOVA | | | | Univariable - adonis | | | | Multivariable - PERMANOVA | | | |
|  | Df | R² | F | *P* value | Df | R² | F | *P* value | Df | R² | F | *P* value | Df | R² | F | *P* value |
| **Asthma** | 1 | 0.00046 | 0.774 | 0.537 | 1 | 0.00033 | 0.580 | 0.747 | 1 | 0.00108 | 1.767 | 0.131 | 1 | 0.00073 | 1.304 | 0.239 |
| **Sex** | 1 | 0.02370 | 39.57 | 0.001 | 1 | 0.02266 | 40.19 | 0.001 | 1 | 0.02370 | 39.568 | 0.001 | 1 | 0.02261 | 40.137 | 0.001 |
| **Age** | 1 | 0.00304 | 4.964 | 0.005 | 1 | 0.00178 | 3.162 | 0.019 | 1 | 0.00304 | 4.964 | 0.003 | 1 | 0.00179 | 3.178 | 0.020 |
| **Ethnicity** | 5 | 0.05393 | 18.54 | 0.001 | 5 | 0.03116 | 11.06 | 0.001 | 5 | 0.05393 | 18.536 | 0.001 | 5 | 0.03115 | 11.057 | 0.001 |
| **BMI** | 1 | 0.00883 | 14.51 | 0.001 | 1 | 0.00590 | 10.47 | 0.001 | 1 | 0.00883 | 14.514 | 0.001 | 1 | 0.00589 | 10.458 | 0.001 |
| **Smoking status** | 2 | 0.00401 | 3.282 | 0.003 | 2 | 0.00193 | 1.709 | 0.076 | 2 | 0.00401 | 3.232 | 0.005 | 2 | 0.00193 | 1.714 | 0.086 |
| **Alcohol use** | 1 | 0.00838 | 6.881 | 0.001 | 1 | 0.00113 | 1.005 | 0.363 | 1 | 0.00838 | 6.881 | 0.001 | 1 | 0.00115 | 1.020 | 0.377 |
| **Total fatty acids** | 1 | 0.00457 | 7.489 | 0.001 | 1 | 0.00046 | 0.818 | 0.478 | 1 | 0.00457 | 7.489 | 0.001 | 1 | 0.00044 | 0.788 | 0.524 |
| **Fibres** | 1 | 0.00420 | 6.868 | 0.001 | 1 | 0.00108 | 1.917 | 0.082 | 1 | 0.00420 | 6.868 | 0.002 | 1 | 0.00106 | 1.886 | 0.089 |
| **Saturated fatty acids** | 1 | 0.00411 | 6.719 | 0.001 | 1 | 0.00034 | 0.601 | 0.705 | 1 | 0.00411 | 6.719 | 0.001 | 1 | 0.00033 | 0.589 | 0.731 |
| **Recent antibiotics** | 1 | 0.00226 | 1.849 | 0.061 | 1 | 0.00193 | 1.710 | 0.054 | 1 | 0.00226 | 1.849 | 0.054 | 1 | 0.00192 | 1.707 | 0.067 |
| **Use of probiotics** | 1 | 0.00151 | 1.232 | 0.259 | 1 | 0.00062 | 0.549 | 0.901 | 1 | 0.00151 | 1.232 | 0.250 | 1 | 0.00062 | 0.549 | 0.902 |
| **Use of corticosteroids** | 1 | 0.00064 | 1.047 | 0.326 | 1 | 0.00052 | 0.925 | 0.431 | 1 | 0.00064 | 1.047 | 0.348 | 1 | 0.00055 | 0.974 | 0.336 |
| **Season of sample collection** | 3 | 0.00369 | 2.010 | 0.034 | 3 | 0.00280 | 1.657 | 0.066 | 3 | 0.00369 | 2.010 | 0.024 | 3 | 0.00281 | 1.663 | 0.070 |

Analyses were performed by permutational multivariate analysis of variance (PERMANOVA) with the Weighted UniFrac distance. Two alternative definitions for asthma were used: (1) participants were considered asthmatics based on asthma medication usage (strict definition), or (2) based on self-reported symptoms, medication usage and/or doctors’ diagnosis (broad definition). The multivariable model includes sex, age, ethnicity, BMI, smoking status, alcohol consumption, dietary variables (total fatty acids, saturated fatty acids and fibres), recent use of antibiotics (3 months prior to faecal sample collection), usage of probiotics and corticosteroids, and the season of sample collection.

**Supplementary Table 2. Univariable and multivariable analysis of associations between individual characteristics and gut microbiota β-diversity of asthmatics (n=90) vs. non-asthmatics (n=916) following exclusion of participants with comorbidities**

|  | **Weighted Unifrac** | | | | **Weighted Unifrac** | | | |  |
| --- | --- | --- | --- | --- | --- | --- | --- | --- | --- |
|  | Univariable - adonis | | | | Multivariable - PERMANOVA | | | |  |
|  | Df | R² | F | *P* value | Df | R² | F | *P* value | Contribution to total inter-individual dissimilarities |
| **Asthma** | 1 | 0.00058 | 0.587 | 0.709 | 1 | 0.00041 | 0.452 | 0.847 | 0.53 % |
| **Sex** | 1 | 0.02337 | 24.02 | 0.001 | 1 | 0.02208 | 24.13 | 0.001 | 28.27 % |
| **Age** | 1 | 0.00181 | 1.825 | 0.100 | 1 | 0.00159 | 1.738 | 0.112 | 2.04 % |
| **Ethnicity** | 5 | 0.05651 | 11.98 | 0.001 | 5 | 0.03305 | 7.225 | 0.001 | 42.31 % |
| **BMI** | 1 | 0.01047 | 10.62 | 0.001 | 1 | 0.00383 | 4.184 | 0.009 | 4.90 % |
| **Smoking status** | 2 | 0.00727 | 3.675 | 0.003 | 2 | 0.00348 | 1.900 | 0.065 | 4.45 % |
| **Alcohol use** | 1 | 0.01322 | 13.45 | 0.001 | 1 | 0.00098 | 1.073 | 0.319 | 1.26 % |
| **Total fatty acids** | 1 | 0.00415 | 4.183 | 0.004 | 1 | 0.00144 | 1.569 | 0.150 | 1.84 % |
| **Fibers** | 1 | 0.00355 | 3.573 | 0.013 | 1 | 0.00194 | 2.121 | 0.071 | 2.48 % |
| **Saturated fatty acids** | 1 | 0.00310 | 3.123 | 0.014 | 1 | 0.00094 | 1.025 | 0.344 | 1.20 % |
| **Recent antibiotics** | 1 | 0.00255 | 1.283 | 0.205 | 1 | 0.00207 | 1.133 | 0.302 | 2.66 % |
| **Use of probiotics** | 1 | 0.00249 | 1.251 | 0.237 | 1 | 0.00136 | 0.742 | 0.669 | 1.74 % |
| **Use of corticosteroids** | 1 | 0.00078 | 0.785 | 0.534 | 1 | 0.00129 | 1.414 | 0.177 | 1.66 % |
| **Season of sample collection** | 3 | 0.00400 | 1.343 | 0.172 | 3 | 0.00365 | 1.329 | 0.176 | 4.67 % |
| **Total** |  |  |  |  |  | 0.07810 |  |  | 100 % |

Participants with the following comorbidities were excluded: diabetes mellitus, hypertension, cardiovascular disease, chronic gastrointestinal disease, malignancy, stroke and rheumatic disease. Analyses were performed by permutational multivariate analysis of variance (PERMANOVA) with the Weighted UniFrac distance. The multivariable model includes sex, age, ethnicity, BMI, smoking status, alcohol consumption, dietary variables (total fatty acids, saturated fatty acids and fibers), recent use of antibiotics (3 months prior to fecal sample collection), usage of probiotics and corticosteroids, and the season of sample collection. *Df*: degrees of freedom.

**Supplementary Figure 1**

**Supplementary Figure 1. Core fecal microbiota composition of asthmatics (n=172) and non-asthmatics (n=1460)**

Core microbiota heatmaps showing the relative abundance and prevalence of phyla (A) or families (B) across samples from asthmatics (left) and non-asthmatics (right). Phyla and families that are present in at least 25% of the participants are shown.


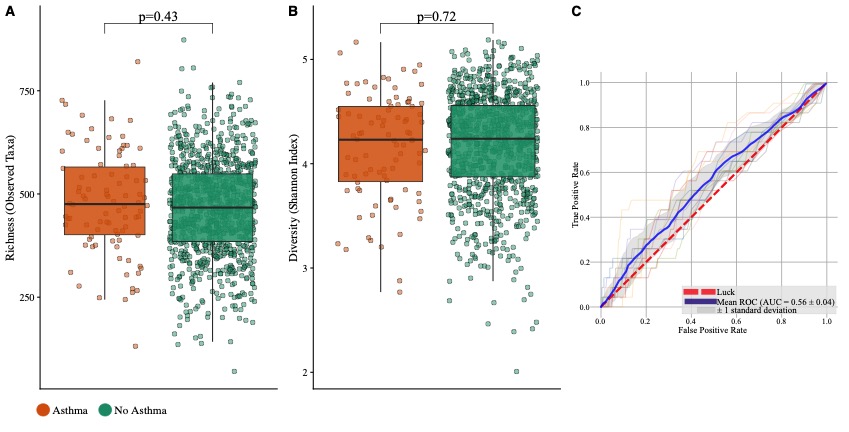
**Supplementary Figure 2**

**Supplementary Figure 2. No prominent differences in fecal microbiota composition between asthmatics and non-asthmatics following exclusion of participants with comorbidities**

No differences in Shannon diversity (A) and richness (B) between adult asthmatics (n=90) and non-asthmatics (n=916). Receiver operating characteristic curve of the extremely randomized trees classifier showing asthmatics could not be distinguished from non-asthmatics based on individual microbiota composition (C). Participants with the following comorbidities were excluded: diabetes mellitus, hypertension, cardiovascular disease, chronic gastrointestinal disease, malignancy, stroke and rheumatic diseases.

In the box plots the central line shows the median, the box limits are the first and third quartile, and whiskers above and below the box. ROC = receiver operating characteristic; AUC = area under the curve.

**Supplementary Figure 3**

**
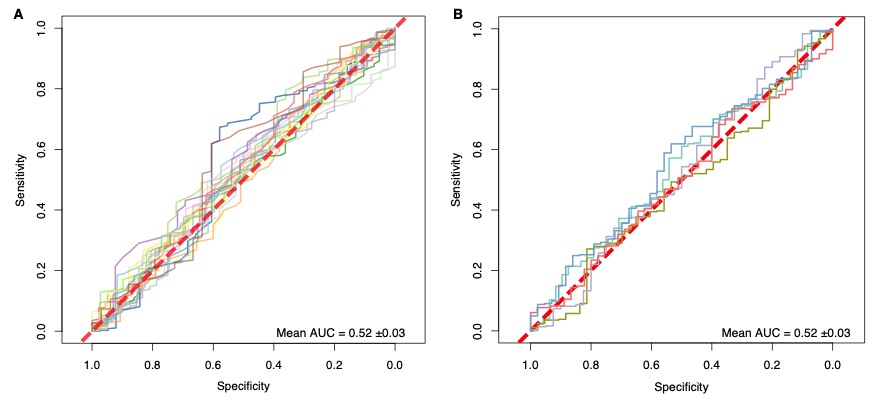
**

**Supplementary Figure 3. No discriminating capacity of two additional machine learning models to distinguish asthmatics from non-asthmatics**

Receiver operating characteristic curve of a random forest model (A) and Support Vector Machines (B) showing asthmatics could not be distinguished from non-asthmatics based on individual microbiota composition. AUC = area under the curve.

**Supplementary Figure 4**

**
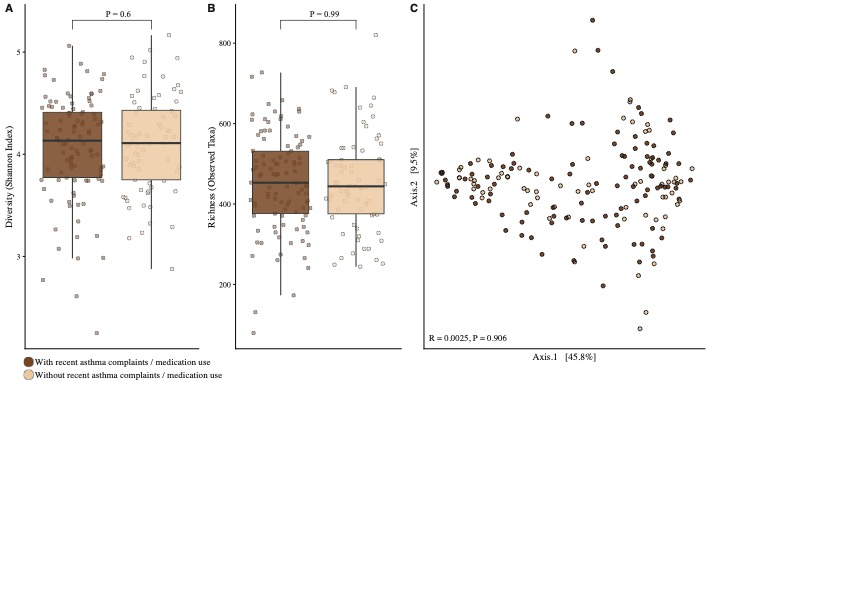
**

**Supplementary Figure 4. No major relationship between reported control of asthma symptoms and microbiota richness or diversity.** Comparison of Shannon α-diversity (A), richness (B) and β-diversity with weighted Unifrac distance (C) between adult asthmatics with (n=101), versus without (n=71) complaints or use of asthma medication in the past year. In the box plots the central line shows the median, the box limits are the first and third quartile, and whiskers above and below the box.
